# Therapeutic Effect of Nicotinamide Mononucleotide for Hypoxic–Ischemic Brain Injury in Neonatal Mice

**DOI:** 10.1177/17590914231198983

**Published:** 2023-10-03

**Authors:** Takuya Kawamura, Gagandeep Singh Mallah, Maryam Ardalan, Tetyana Chumak, Pernilla Svedin, Lina Jonsson, Seyedeh Marziyeh Jabbari Shiadeh, Fanny Goretta, Tomoaki Ikeda, Henrik Hagberg, Mats Sandberg, Carina Mallard

**Affiliations:** 1 Institute of Neuroscience and Physiology, Centre of Perinatal Medicine and Health, 70712 Sahlgrenska Academy, 70712 University of Gothenburg, Gothenburg, Sweden; 2 Department of Obstetrics and Gynecology, 12946 Mie University, Tsu, Japan; 3 Centre of Perinatal Medicine and Health, Institute of Clinical Sciences, Gothenburg, Sweden; 4 Institute of Biomedicine, 70712 Sahlgrenska Academy, 195564 University of Gothenburg, Gothenburg, Sweden

**Keywords:** sirtuins, alarmins, infant, therapy, hypoxic–ischemic encephalopathy, nicotinamide adenine dinucleotide

## Abstract

A clinical challenge remains in the treatment of hypoxic–ischemic brain injury in newborns. Nicotinamide adenine dinucleotide (NAD^+^) has beneficial effects in animal models of adult stroke. Here, we aimed to understand the short- and long-term neuroprotective effects of NAD^+^-promoting substance nicotinamide mononucleotide (NMN) in a well-established brain injury model in neonatal mice. Postnatal day (PND) 9 male and female mice were subjected to cerebral hypoxia–ischemia and treated with saline or NMN (50 mg/kg) immediately after hypoxia–ischemia. At different time points after hypoxia–ischemia, hippocampal NAD^+^, caspase-3 activity, protein expression of SIRT1, SIRT6, release of high mobility group box-1 (HMGB1), long-term neuropathological outcome, short-term developmental behavior, and long-term motor and memory function were evaluated. Neonatal hypoxia–ischemia reduced NAD^+^ and SIRT6 levels, but not SIRT1, in the injured hippocampus, while HMGB1 release was significantly increased. NMN treatment normalized hippocampal NAD^+^ and SIRT6 levels, while caspase-3 activity and HMGB1 release were significantly reduced. NMN alleviated tissue loss in the long-term and improved early developmental behavior, as well as motor and memory function. This study shows that NMN treatment provides neuroprotection in a clinically relevant neonatal animal model of hypoxia–ischemia in mice suggesting as a possible novel treatment for neonatal brain injury.

**Summary Statement:**

- Neonatal hypoxia–ischemia reduces nicotinamide adenine dinucleotide (NAD^+^) and SIRT6 levels in the injured hippocampus.
- Hippocampal high mobility group box-1 (HMGB1) release is significantly increased after neonatal hypoxia–ischemia.
- Nicotinamide mononucleotide (NMN) treatment normalizes hippocampal NAD^+^ and SIRT6 levels, with significant decrease in caspase-3 activity and HMGB1 release.
- NMN improves early developmental behavior, as well as motor and memory function.

## Graphical abstract

**Graphical Abstract:**
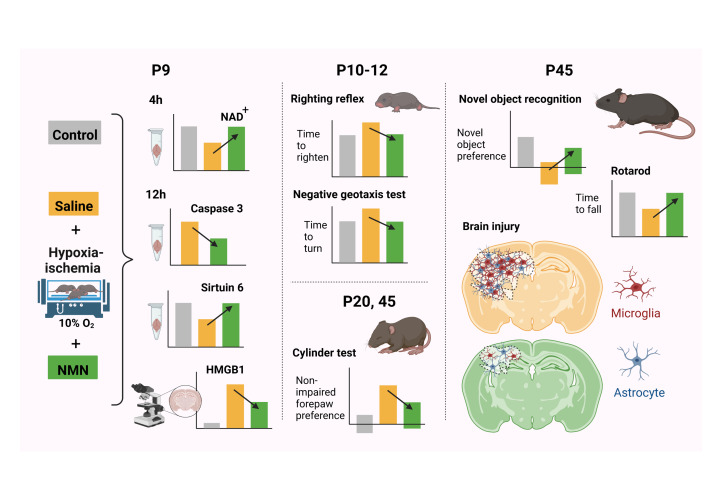
This is a visual representation of the abstract.

## Introduction

Hypoxic–ischemic encephalopathy (HIE) has an incidence of 1 to 3 per 1,000 live births and is one of the leading causes of infant death, mental retardation, seizures, and cerebral palsy (Kurinczuk et al., 2010; Vexler and Ferriero, 2001). Neonatal hypoxic–ischemic (HI) brain injury evolves over days and possibly weeks, providing a therapeutic window (Ferriero, 2004). Therefore, understanding the cellular pathways underlying HI may provide new approaches to HI treatment in newborns. Nicotinamide adenine dinucleotide (NAD^+^) is an essential cofactor for multiple cellular metabolic reactions and has a crucial role in energy production. Mitochondrial dysfunction has been demonstrated to be an important factor for the development of secondary brain injury after HI (Hagberg et al., 2014). Preservation of NAD^+^ following adult stroke reduces ischemic infarct size (Liu et al., 2009). NAD^+^ can be generated in cells either by de novo synthesis from tryptophan or resynthesized from nicotinamide with nicotinamide phosphoribosyltransferase (NAMPT) as the rate-limiting enzyme (Belenky et al., 2007; Owens et al., 2013). Nicotinamide mononucleotide (NMN) is an enzymatic product of NAMPT (Revollo et al., 2004), and administration of NMN increases NAD^+^ concentration in cells (Belenky et al., 2007) with beneficial effects against cerebral ischemic injury in the adult mouse brain (Park et al., 2016) and HI rat pups (Feng et al., 2006) but has not been studied in neonatal HI in mice.

Sirtuins (SIRT) belong to a family of NAD-dependent class III histone deacetylases (HDAC) (Imai et al., 2000; Landry et al., 2000) where SIRT1 and SIRT6 are nuclear proteins (She et al., 2017). SIRT1 overexpression is protective in adult stroke models (Hattori et al., 2014), while SIRT1-deficient mice display larger infarct volume after permanent middle cerebral artery occlusion (MCAO) (Hernandez-Jimenez et al., 2013). In a mouse study, vascular SIRT6 expression was reduced following stroke and overexpression of SIRT6 decreased infarct size and neurological deficits (Liberale et al., 2020). *In vitro* studies demonstrated that reduced neuronal SIRT6 expression following oxygen/glucose deprivation induces release of alarmin high mobility group box-1 (HMGB1) from cell nuclei (Lee et al., 2013). HMGB1 is a ubiquitous nonhistone DNA-binding protein located in the nucleus that functions as a structural cofactor to regulate transcription (Ulloa & Messmer, 2006). Under physiological conditions, HMGB1 induces neurite growth in the immature brain (Kim et al., 2008; Merenmies et al., 1991). However, under pathological conditions, HMGB1 is released from the nucleus into the extracellular space of activated immune cells and acts as a pro-inflammatory mediator (Wang et al., 1999). HMBG1 nuclear translocation has also been observed in injured cells and in animal models of adult stroke (Kim et al., 2006; Zhang et al., 2011) and neonatal HI brain injury (Chen et al., 2019). Inhibition of nuclear translocation of HMGB1 was recently shown to reduce neurological injury and improve neurobehavioral impairments following neonatal HI (Le et al., 2020).

Therapeutic hypothermia is currently used as a clinical treatment for HIE and improves survival and reduces neurological sequelae yet is not effective in all infants (Azzopardi et al., 2014; Gluckman et al., 2005), and additional treatment strategies are needed. Although there is evidence of protective effects of NMN on cerebral ischemia in adults (Park et al., 2016) and reduced brain weight deficits in HI rat pups (Feng et al., 2006), research to date has not investigated the neuroprotective potential of NMN in neonatal brain injury in mouse. Therefore, the present study was designed to investigate cellular neuroprotective mechanisms of NMN by focusing on SIRT1 and SIRT6 expressions, HMGB1 nuclear translocation, and parameters of neuroinflammation and astrogliosis, as well as motor and memory function, in a well-established model of HI in neonatal mice.

## Materials and Methods

The study designed is shown in Supplemental Figure 1.

### Animals

C57BL/6J mice were bred in-house at the laboratory of Experimental Biomedicine at Sahlgrenska Academy, University of Gothenburg, Sweden, under standard conditions: 12-h light/dark cycle, ad libitum access to standard laboratory chow diet (B&K, Solna, Sweden), and drinking water in a temperature-controlled environment (20–22 °C). All animal experiments and methods for animal euthanasia were approved by the Animal Ethics Committee in Gothenburg (No 633/2017) and adhered to best practice (ARRIVE and PREPARE international guidelines) and the European Council directive (2010/63/EU). All personnel working with animals were highly trained and qualified according to Swedish law (L 150, SJVFS 2019:9).

### Hypoxia–Ischemia

Nine-day-old mice of each sex were exposed to HI in the modified Vannucci model (Rice et al., 1981; Hedtjarn et al., 2002). Briefly, anesthesia was induced and maintained with isoflurane gas (5% and 3% respectively in a 1:1 mixture of air and O_2_). The left common carotid artery was permanently ligated with 6.0 silk suture, the incision was closed, and local xylocaine was applied. Following surgery, pups were returned to their mothers for 1 h recovery and then exposed to 10% O_2_ in N_2_ for 50 min in a heated chamber (35.5–36.5 °C). Subsequently, pups stayed with their mothers until sacrifice.

### Administration of Nicotinamide Mononucleotide

Nicotinamide mononucleotide (NMN, Merck, N3501, Lot#SLBX2999, Darmstadt, Germany) was prepared in sterile saline and administered to mice in doses of 50, 150, and 300 mg/kg. NMN doses were selected based on a previous adult stroke study (Park et al., 2016). The pups were randomly allocated to NMN treatment or saline groups within each litter. Drug (NMN) or vehicle (saline) solutions (10 µl/gram body weight) were injected intraperitoneally immediately after HI. Naïve animals were sacrificed without prior surgery, hypoxia, or injections.

### High-Performance Liquid Chromatography for Detecting NAD^+^

To investigate whether NMN restored NAD^+^ concentration after neonatal HI, high-performance liquid chromatography (HPLC) was performed as previously described (Li and Sauve, 2015). The hippocampus from each hemisphere was dissected 4 h after HI and immediately snap-frozen in liquid nitrogen. The frozen samples were weighted and homogenized (1:100 weight/volume) in cold 7% HClO_4_ (70% HClO_4_ (11.65 M) diluted in HPLC-graded water) and then centrifuged at 4 °C for 5 min. The pellet was dissolved in 2 M NaOH and used for protein determination by the bicinchoninic acid protein assay (BCA) method. The supernatants were transferred to new tubes and 1 M K2HPO4 was added to reach to pH 7. The tubes were centrifuged, and the supernatant was used for NAD^+^ determination by HPLC. NAD^+^ (Merck, N0632, Lot#SLBQ5270 V, Darmstadt, Germany) was dissolved in 0.05 M phosphate buffer and used to prepare NAD^+^ standards. The NAD^+^ peak was identified by the addition of known amounts of NAD^+^ to samples. Quantification of NAD^+^ in samples was performed using external standards, that is, the peak area of NAD^+^ absorbance at 254 nm in samples was compared to a linear calibration curve of peak areas of four concentrations of NAD^+^ (0.5−16 μM).

All chromatography was performed using a Varian Prostar HPLC pump coupled to a UV detector (Applied Biosystems 757). Sample injection was made using a Waters 717 autosampler. All separations were performed at room temperature (RT). Data were processed with Millennium32 software (Waters Corporation, Milford, MI, USA). NAD^+^ was separated from other nucleotides on a Nucleosil C18 column (200 × 4.6 mm; 5 μm particle size; HiChrom, UK) fitted with a guard column. The mobile phase consisted of phosphate buffer (50 mM, pH 7) and methanol (Ratburn, UK) 95/5. A flow rate of 1 ml/min was used and peaks were detected by absorbance at 254 nm.

### Tissue Preparation for Caspase-3 Activity Assay and Western Blotting

The hippocampus from each hemisphere was dissected 12 h after HI. The tissues were immediately frozen on dry ice and stored at −80 °C. The tissues were then homogenized in a solution containing: Tris-HCl (25 mM, pH 7.9), NaCl (100 mM), ethylenediaminetetraacetic acid (5 mM), NP40 (1%), sodium butyrate (0.1 mM), Nam (5 mM), protease inhibitor (1%), phosphatase inhibitor, and phosphate buffered saline (PBS) (pH 7.4). Afterwards, samples were centrifuged 15,000*g* for 5 min at 4 °C. Protein concentration was measured by a BCA (QPBCA, Thermo Fisher Scientific, Waltham, MA, USA).

### Caspase-3 Activity Assay

A fluorometric assay for caspase-3 activity was performed on the hippocampal tissue as previously described (Wang et al., 2001). Briefly, a peptide substrate, Ac-Asp-Glu-Val-Asp-aminomethyl coumarine (Ac-DEVO-AMC; #SAP3171v, Peptide Int., Louisville, KY, USA), was blended with tissue samples of the hippocampus. Cleavage of the substrate was measured at 37 °C with a Spectramax Gemini microplate fluorometer (Molecular Devices, Sunnyvale, CA, USA) and an excitation/emission wavelength of 360/460 nm. A 2-min interval was used to track degradation, and the V-max was calculated. Standard curves with 7-amino-4-methyl-coumarin (AMC) were utilized to express the data in picomoles of AMC formed per min and per milligram of protein.

### Western Blotting

Sample lysates of the hippocampus were blended with 4× Laemmli sample buffer (Bio-Rad laboratories, Hercules, CA, USA) and 10% β-mercaptoethanol (Merck, Darmstadt, Germany) and diluted in PBS to equalize their protein concentration and heated (95 °C) for 5 min. Total protein (20 µg/well) was loaded on 4–20% Criterion TGX Stain-Free Precast Gels (Bio-Rad laboratories, Hercules, CA, USA) and transferred onto 0.2 µm nitrocellulose membranes (Bio-Rad laboratories, Hercules, CA, USA). Membranes were blocked in TBS-T (pH 7.45, Tris-Base, NaCl, 0.1% Tween 20) containing 5% nonfat milk for 60 min and then incubated in the following primary antibodies overnight at 4°C: SIRT1 mouse monoclonal antibody (1:500 dilution, CST 8469, Cell Signaling, Leiden, WZ, Netherlands) and SIRT6 rabbit monoclonal antibody (1:500 dilution, CST 12486, Cell Signaling, Leiden, WZ, Netherlands). The following day, membranes were placed with secondary antibody (1:5,000, Vector Laboratory, Burlingame, CA, USA) in 5% nonfat milk for 1 h and the bands were visualized using a Clarity Western ECL substrate (Bio-Rad laboratories, Hercules, CA, USA) and a Gel Doc XR Plus system (Bio-Rad laboratories, Hercules, CA, USA). Immunoreactive bands were quantified using Image Lab Software (Bio-Rad laboratories, Hercules, CA, USA) and normalized to total protein.

### Behavioral Studies

#### Behavioral tests of motor function

Negative geotaxis and surface righting tests, the cylinder test, and rotarod behavioral tests were used to investigate the therapeutic effect of NMN on motor function after the HI insult.

**Righting Reflex**. The righting reflex test was performed as described before (Feather-Schussler & Ferguson, 2016). Briefly, pups were placed in a supine position on a flat stage. The time required to turn over on all four feet and touch the platform was recorded. Each animal was allowed three attempts, with a 5-min interval between. The average value was calculated for each time point and used for further analysis. Assessments were performed at 24, 48, and 72 h after HI insult.
**Negative Geotaxis.** The negative geotaxis test examines the ability of pups to right themselves on an angled surface as described (Feather-Schussler & Ferguson, 2016). Pups were placed head down on a slant fabric surface inclined at an angle of 45°, and the time needed to rotate 180° (head up) was recorded. Each animal was allowed two attempts and the average value was calculated. The test was performed at 24, 48, and 72 h after HI immediately after the righting reflex test.
**Cylinder Rearing Test (CRT).** The CRT was performed as previously described with some modifications (Gustavsson et al., 2005). Briefly, at P20 and P45 ± 5, mice were placed in a transparent glass cylinder (75 mm ×150 mm height and 95 mm ×180 mm height, respectively) and recorded for 3 min by a video camera. The animals were observed to determine their preference for their forepaws during full rearing (left/right/both). The preference proportion (%) of left (nonimpaired) forepaw contact was calculated as follows:
(left−right)/(right+left+both)×100
If the number of total contacts with the glass wall was less than four, the mouse was excluded from the analysis.
**Accelerated Rotarod Test.** The accelerated rotarod test was performed to evaluate motor coordination and performance at P45 ± 5. Animals were placed on the apparatus (Panlab, LE8505, Barcelona, Spain) without rotation for 2 min to accommodate and then with accelerated rotation for four trials with rod acceleration from 0 to 40 rpm over a 5-min period. Animals were allowed 15-min rest between trials. The average time interval from start to the moment when the animal fell off the rod from four trials was calculated.

#### Behavioral test for memory function

**Novel Object Recognition Test.** The novel object recognition test (NORT) was performed to evaluate memory function at P45 ± 5. A slightly modified protocol from that previously described was used (Leger et al., 2013). All experiments were performed during the day between 9 am and 3 pm. The test included three phases: habituation, familiarization, and test phase. The habituation phase was conducted 24 h before the testing day. On day 1 (habituation phase), mice were moved to the behavior testing room and allowed 60-min habituation and then placed in the open field apparatus (50 cm × 50 cm × 50 cm) for 10 min. Thereafter, mice were returned to the holding cage, and the apparatus was cleaned with 70% ethanol to remove olfactory cues.

On day 2, animals were first allowed 60-min habituation in home cage. During the familiarization phase, mice were placed in the apparatus for familiarization (10 min) with two identical objects (A + A) placed in the center of the open field box. The inclusion criteria were that the mouse explored at least one object for at least 20 s in total during the familiarization phase. If the mouse did not explore the object for 20 s in total, the mouse was excluded from the study.

Thereafter, the test was performed using one identical object to the one used in the familiarization phase and one novel object (A + B), for short- (10 min) and long- (3 h) term memories (Antunes and Biala, 2012). For the test phase, the exploration time for the familiarization object and the novel object was calculated separately (Sivakumaran et al., 2018). All steps in the experiment were video recorded and analyzed manually by a researcher blinded to the experimental groups. Exploration was defined as touching an object with the nose or forepaws. Climbing onto the object or being on top of the object did not qualify as exploration. The discrimination index (DI) was used as an indicator of memory function (short-term and long-term) and calculated as:

$$DI=\frac{T_{\left(\mathrm{new}\right)}-T_{\left(\mathrm{familiar}\right)}}{T_{\left(\mathrm{total}\right)}}$$

*T*_new_ is the exploring time with novel object; *T*_familiar_ is the exploring time with familiar object; *T*_total_ is the total exploring time. This index can vary between +1 and −1, where a positive score indicates more time spent with the novel object, a negative score indicates more time spent with the familiar object, and a zero indicates no preference (Antunes & Biala, 2012).

### Tissue Processing and Immunohistochemistry for HMGB1

Mice were euthanized via intraperitoneal administration of pentobarbital (Pentacour) (60 mg/ml) 12 h after HI, and brains were removed and immersed in Histofix (Histolab, Västra Frölunda, Sweden) for 14 days and then placed in 30% sucrose for 48 h followed by snap-freezing in isopentane (Sigma Aldrich). Brains were cut into 70-μm-thick coronal sections on a cryostat (Leica CM 3050S). Series of sections were collected based on a systematic sampling principle, with a section sampling fraction of one out of six sections (1/6). One set of sections was used for HMGB1 immunostaining. Free-floating sections were washed in PBS (0.1 M, pH 7.2, 30 min) followed by antigen retrieval using 0.01 M citrate buffer (40 min at 85 °C). In the next step, sections were washed in 0.25% triton followed by blocking with endogenous peroxidase (3% H_2_O_2_ in PBS) for 15 min. Thereafter, sections were rinsed in PBS (2 × 15 min) and placed in 4% goat serum for 1 h. In the next step, sections were incubated with a monoclonal rabbit anti-HMGB1 (1:350, Abcam, Ref# ab79823, Cambridge, UK, RRID:AB_1603373) overnight at 4 °C. Subsequently, sections were washed in PBS and then incubated in polyclonal secondary goat anti-rabbit IgG (1:200 dilution; Dako, Ref# P0448, Denmark) for 2 h followed by washing in PBS (2 × 15 min) and ABC elite (avidin–biotin complex, Vector laboratory, Burlingame, CA, USA) for 1 h. Color labeling was performed using 3,3-diaminobenzidine (DAB) solution (Sigma, USA). Finally, the sections were mounted on gelatin-coated slides and dehydrated through a graded series of alcohol (95%, 99%), cleared in xylene, and coverslipped.

### Analysis of HMGB1-Positive Cell Number Density in the Hippocampus

To test the effect of NMN on hippocampal HMGB1 nuclear translocation, we quantified the number of HMGB1-positive cells in two subregions of the hippocampus (CA1 pyramidal cell layer (CA1.P) and CA3 pyramidal cell layer (CA3.P)) in both ipsi- and contralateral hippocampi 12 h after HI. HMGB1-positive cells were classified into two types: nuclear HMGB1-positive (only HMGB1-positive nuclear staining; Figure 2C, black arrow) and cytoplasmic HMGB1-positive (less nuclear staining intensity as well as presence of cytosolic HMGB1 staining; Figure 2C, red arrow) (Chen et al., 2019). Delineation of the regions of interest (CA1.P and CA3.P) was performed using 5× objective lens. To avoid the effect of shrinkage and tissue loss on the number of counted cells, cell density measurements were performed on two randomly systematically selected sampling frames from three sections per animal (in total six sampling frames per animal) with a 100× oil immersion objective lens using optical probe with the height of 10 μm. The counting frame size was 854.33 µm^2^. The number density of cells was calculated by applying the following formula (Ardalan et al., 2022):

Nv=∑Q−/V

where *N*_v_ indicates the number of cells per volume of sampling brain region, Σ*Q*- is the number of cells counted, and *V* is the volume of sampled regions of interest.

**Figure 1.**
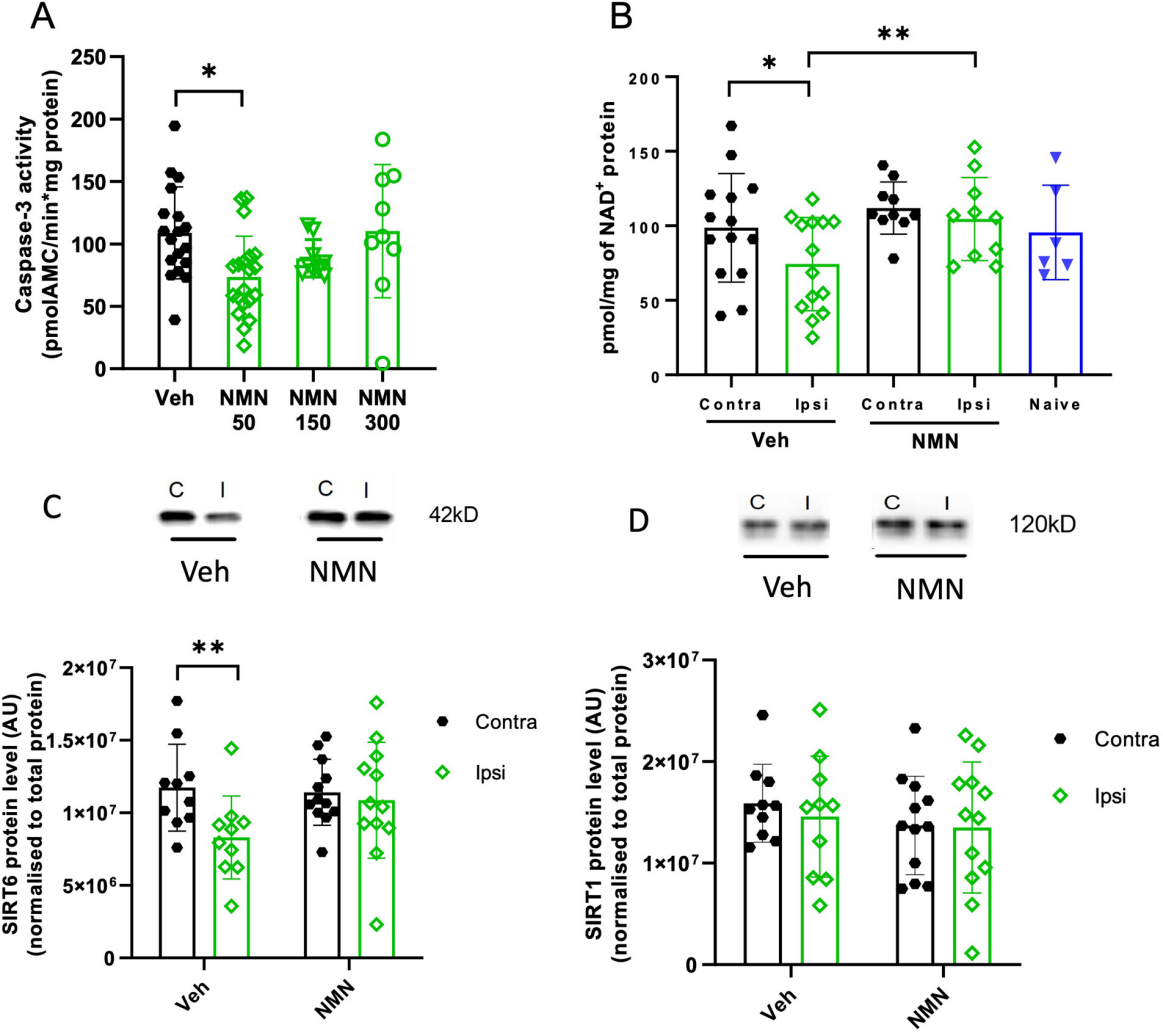
NMN effects on caspase-3 activity, NAD^+^ levels, and SIRT1 and SIRT6 expression in hippocampus after neonatal HI. Postnatal day 9 pups were exposed to 50 min hypoxia–ischemia (HI). (A) Pups were allocated to four treatment groups: NMN 50 mg/kg, 150 mg/kg, and 300 mg/kg and vehicle (n = 9–20/grp). All drugs were injected immediately after HI by intraperitoneal administration and caspase-3 activity measured in the hippocampus 12 h later. (B) NAD^+^ was measured by high-performance liquid chromatography in the ipsilateral (ipsi) and contralateral (contra) hippocampus 4 h after HI in vehicle and NMN 50 mg/kg (n = 10–13/grp) and in naïve P9 mice (n = 6). Protein expression for SIRT1 (C) and SIRT6 (D**)** was measured by Western blotting in the ipsilateral and contralateral hippocampus 12 h after HI, with NMN 50 mg/kg or without NMN treatment (n = 10–12/grp). Data presented as mean ± SD. *p < .05, **p < .01.

The percentage of cytoplasmic HMGB1-positive cells for each hippocampal region from ipsi- and contralateral side was calculated (*N*_v_ cytoplasmic HMGB1-positive cells/*N*_v_ of HMGB1-positive cells × 100).

### Tissue Processing and Immunohistochemistry for Microtubule-Associated Protein 2, Ionized Calcium-Binding Adaptor Molecule, and Glial Fibrillary Acidic Protein

At P45 ± 5, mice were deeply anesthetized via intraperitoneal injection of pentobarbital (Pentacour, 60 mg/ml). Brains were removed and placed in Histofix for 2 weeks and then paraffin embedded and cut into 15-µm-thick coronal sections with a systematic sampling fraction of 1/20. Microtubule-associated protein 2 (MAP-2), ionized calcium-binding adaptor molecule (Iba1), and glial fibrillary acidic protein (GFAP) staining was performed as described previously (Svedin et al., 2007). Briefly, sections were deparaffinized, hydrated in xylene and graded alcohol, and boiled in citric acid buffer (0.01 M, pH 6.0, 30 min), followed by blocking of endogenous peroxidase and nonspecific antibody binding using 4% goat serum in PBS (0.1 M, pH 7.2). In the next step, after washing in PBS, sections were incubated with mouse monoclonal anti-MAP-2 (clone HM-2, 1:1,000; Sigma Aldrich, St. Louis, Missouri, USA), polyclonal rabbit anti-Iba1 (Cat# 019–19741; 1:2,000; Fujifilm Wako Chemicals U.S.A. Corporation, Richmond, Virginia, USA, RRID:AB_839504), or polyclonal rabbit anti-GFAP (1:500, Dako) overnight at 4°C. The next day, sections were washed in PBS-T and subsequently incubated in polyclonal secondary biotinylated goat anti-rabbit antibody or goat anti-mouse (1:250, Vector Laboratories, Olean, NY, USA) in PBS-T for 2 h in RT. Sections were then washed in PBS-T followed by incubation in ABC elite solution (1.5% solution A + 1.5% solution B in PBS, Vector Laboratories, Burlingame, California, USA) for 1 h at RT. Afterwards, sections were washed in PBS-T for 20 min, and immunolabeling was performed by using 3,3-diaminobenzidine solution (Acros Organics, Geel, Belgium) and enhanced with 15 mg/ml NiSO4. Sections were washed in distilled H_2_O and PBS, dehydrated in 95% and 99% alcohol and xylene, and coverslipped.

### Analysis of Neuropathological Outcome

Gray matter injury was quantified on MAP-2-stained sections as previously described (Svedin et al., 2007). Images of stained sections were captured on a light microscope (Olympus BX60) using a 4× objective lens. The MAP-2 + area was outlined in ipsilateral and contralateral hemisphere (to calculate hemisphere tissue loss) and hippocampus (to calculate hippocampal tissue loss) and measured with ImageJ software (v1.52a, NIH, USA). To calculate the percentage of MAP-2 + hemisphere/hippocampal tissue loss, the MAP-2 + area in the ipsilateral hemisphere/hippocampus was subtracted from the contralateral hemisphere/hippocampus for each brain level and expressed as percentage tissue loss of the contralateral hemisphere/hippocampus:

(contralateral−ipsilateral)/contralateral×100

### Analysis of Microglia and Astrocyte Number Density in the Hippocampus

At age of P45 ± 5 mice, measurement of microglia and astrocyte cell density was performed using light microscopy with a 20× objective lens using the newCAST software. Delineation of the ipsilateral hippocampus and contralateral hippocampus was performed using 5× objective lens. The number of astrocyte and microglia cells was counted when the soma of the cells was in focused. The area sampling fraction (ASF) was 10%. The number density of cells was calculated by applying the following formula:

N=∑Q−/sizeofarea

where *N* indicates the number density of cells and Σ*Q*- is the number of cells counted.

### Measurement of Microglia Soma Volume

The quantification of microglia soma volume was performed using a 3D nucleator under a light microscope with a 100× oil immersion objective lens in the ipsilateral and contralateral hippocampi on Iba1-stained sections (Anzabi et al., 2018). The number of half-lines was set at 6, and the mode was vertical uniform random based on the assumption of rotational symmetry of microglia. For each animal, 50–80 microglia cells were randomly sampled by using the optical disector with 3% ASF and a height of 10 μm.

### Statistics Analysis

Data analysis was performed by a researcher who was blinded to the experimental groups. All data were analyzed using SPSS (IBM corp. released 2013, Version 26.0. Armonk, NY, USA). Graphs were created using prism 8 (GraphPad Software Inc., USA). We calculated the group sizes as inputs and the effect size that the study has (1 − β) power to detect. The effect size was calculated using the T statistic (with a noncentrality parameter): α (two-tailed) = .05, β=.2, S = 1, N0 = 15, N1 = 15, and total group size = N_total_ = N1 + N0 = 30. Proportion of subjects in Group 1 = q1 = N1 / N_total_ = 0.500. Proportion of subjects in Group 0 = q0 = 1 − q1 = 0.500. Degrees of freedom = DoF = N_total_ − 2 = 28. The standard T value (with DoF as degrees of freedom) corresponding to α = Tα = 2.048. k = (1/N1 + 1/N0)1/2 = 0.3651. Noncentrality parameter = δ = 2.9022, E/S = k * δ = 1.0597. The study has 80.0% power to detect an effect size of E = S * E/S = 1.060 (Chow et al., 2017). The statistical analysis assumption for applying parametric tests including normal distribution of data and variance homogeneity of data was checked by making a Q–Q plot of the data and using Levene's test respectively. Comparison of variables between two independent groups (HI-saline and HI/NMN) was performed using independent t-test. Comparison of variables between more than two groups with one independent factor was done by using one-way ANOVA test followed by Tukey post-hoc analysis (equal variances) and Games–Howell (not equal variances) with adjusted *p* values. For analysis between ipsilateral and contralateral sides within the same animal, the data were considered as paired, and paired t-test was applied. Rightening reflex and geotaxis data at different time points (24 h, 48 h, and 72 h) were considered as paired data, and repeated measure ANOVA test was applied. We tested the correlation between variables by doing two-tailed Pearson analysis. In all cases, the significance level was set as *p *< .05. The results are presented as mean ± standard deviation.

## Results

### NMN Attenuates Caspase-3 Activity After Neonatal HI

To evaluate the neuroprotective efficacy dose of NMN, caspase-3 activity was measured in the ipsilateral hippocampus 12 h following HI using either of three doses of NMN (50 mg/kg, 150 mg/kg, and 300 mg/kg). NMN at the dose of 50 mg/kg significantly reduced caspase-3 activity in the ipsilateral hippocampus in the HI group compared to vehicle-treated HI mice (*p *= .021; Figure 1A), and therefore, 50 mg/kg NMN was used for all subsequent experiments.

**Figure 2.**
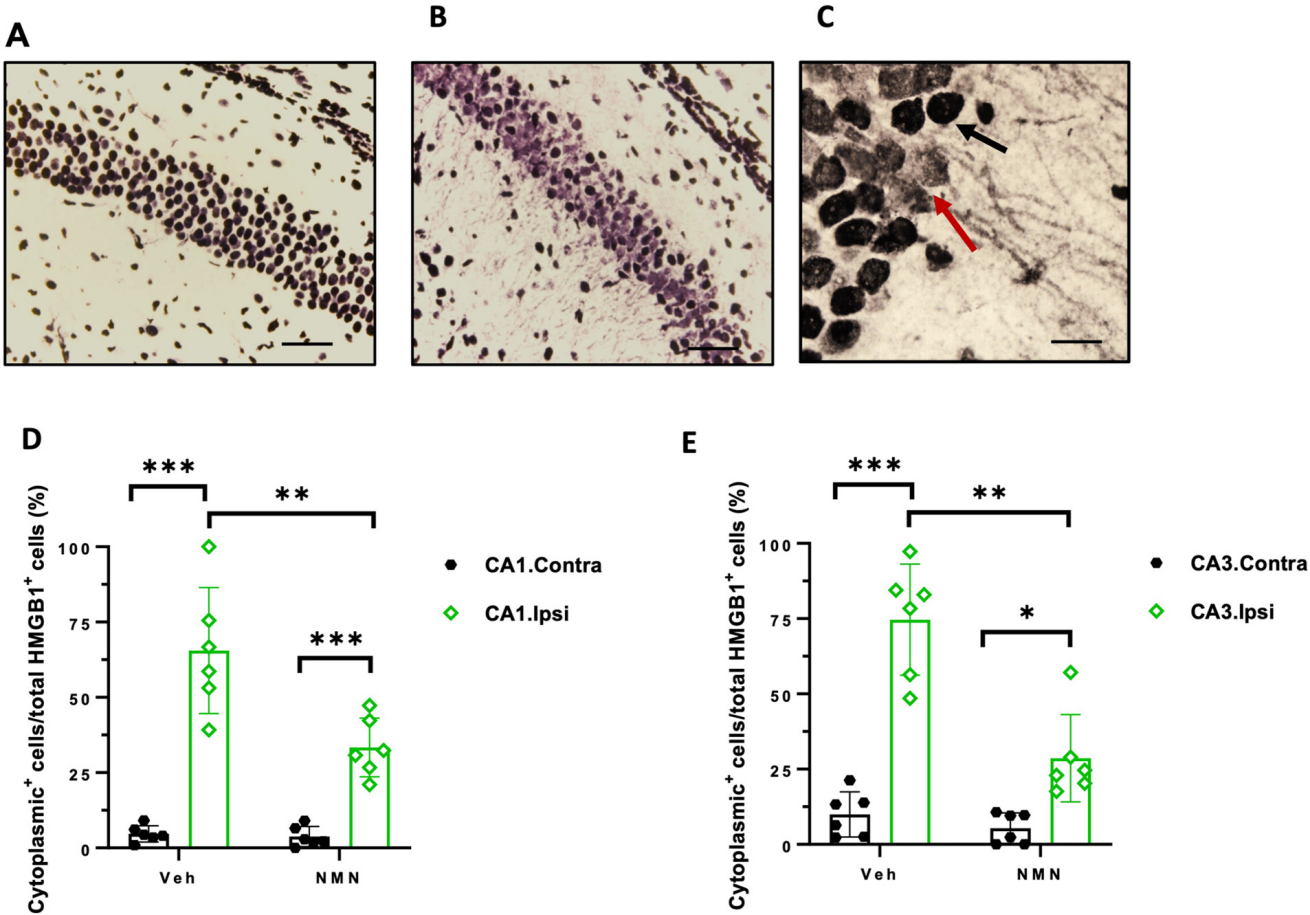
NMN effects on HMGB1 translocation in the hippocampal CA1 and CA3 subregions after neonatal HI. Postnatal day 9 pups were exposed to 50 min hypoxia–ischemia (HI) and brains immunohistochemically stained for HMGB1 12 h after HI in the contralateral (A) and ipsilateral (B) hippocampus. Examples of HMGB1-positive staining within only nuclei (black arrow) and cytoplasmic (lower intensity of nuclear staining along with the presence of cytosolic HMGB1 staining, red arrow) in pyramidal cells in the ipsilateral CA1 subregion of hippocampus (C). The number of cells with cytoplasmic or nuclear HMGB1-positive staining was counted with or without NMN treatment in the contralateral (contra) and ipsilateral (ipsi) CA1 (D) and CA3 (E) subregions of hippocampus (n = 6/grp). Scale bar = 200 µm (A, B) and 20 µm (C). Data presented as mean ± SD. *p < .05, **p < .01, ***p < .001.

### NMN Restores Hippocampal NAD^+^ and Prevents Reduction of Hippocampal SIRT6 but not SIRT1 Protein Expression After Neonatal HI

In vehicle-treated mice, 4 h after HI, ipsilateral hippocampal NAD^+^ concentrations were significantly lower than in the contralateral hippocampus (*p *= .024; Figure 1B). In the NMN-treated group, there was no difference in NAD^+^ concentration between the ipsilateral and contralateral hippocampus (*p *= .447; Figure 1B). Moreover, NMN treatment resulted in a significant increase in ipsilateral hippocampal NAD^+^ concentration compared to the NAD^+^ concentration in the ipsilateral hippocampus of vehicle mice (*p *= .001; Figure 1B).

In the vehicle group, SIRT6 expression in the ipsilateral hippocampus was significantly lower than in the contralateral side (*p *= .004; Figure 1C); however, in NMN-treated mice, SIRT6 expression was similar to the level in the contralateral hippocampus. There was no significant difference in SIRT1 expression between the ipsilateral and contralateral hippocampi in vehicle or NMN treatment groups (Figure 1D).

### NMN Prevents HMGB1 Nuclear Translocation After Neonatal HI

In the contralateral hippocampus, HMGB1-positive staining was observed in the nucleus following HI (Figure 2A), while both nuclear and cytoplasmic staining was evident on the ipsilateral side (Figure 2B and C).

**Figure 3.**
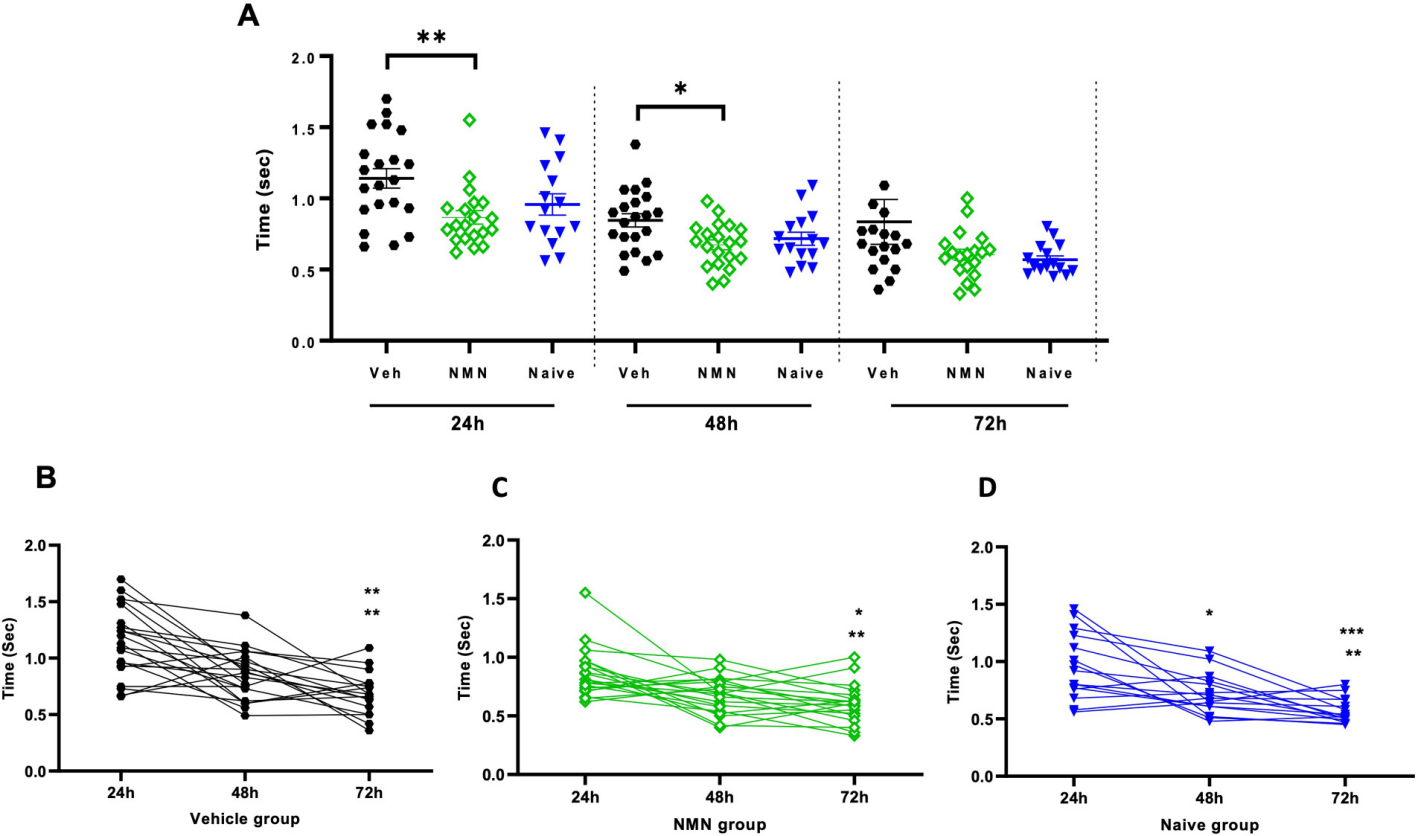
NMN effects on the righting reflex after neonatal HI. Postnatal day 9 pups were exposed to 50 min hypoxia–ischemia (HI) with NMN or without (Veh) treatment. Pups were tested for the righting reflex at 24, 48, and 72 h after HI/NMN or HI/Veh (*n* = 21–18/grp/time point) and in naïve P9-P11 animals (*n* = 15/time point) (A). Within-group pairwise analysis of righting reflex over time in Veh-treated (B) and NMN-treated (C) HI and in naïve (D) animals. Data presented as mean ± SD. *p < .05; **p < .01; ***p < .001.

In the pyramidal layer of both CA1 and CA3 subregions of the ipsilateral hippocampus, the percentages of cells with cytoplasmic location of HMGB1 to the total HMGB1-positive cells were significantly lower in the NMN group compared to vehicle-treated animals (CA1: *p *= .007, CA3: *p *= .001; Figure 2D and E, respectively). There were also significant differences in the percentage of cells with cytoplasmic location of HMGB1 to the total HMGB1-positive cells between the ipsilateral and contralateral sides in CA1.P and CA3.P in both vehicle and NMN groups (CA1: *p *= .000, *p *= .000; CA3: *p *= .000, *p *= .019; Figure 2D and E, respectively). In the contralateral side, the number of cells with HMGB1-positive cytoplasm was very low and we did not observe a difference in the percentage of these cells to the total number of HMGB1-positive cells between the groups in either CA1.P or CA3.P (Figure 2D and E).

### NMN Improves Motor and Memory Function After Neonatal HI

**Figure 4.**
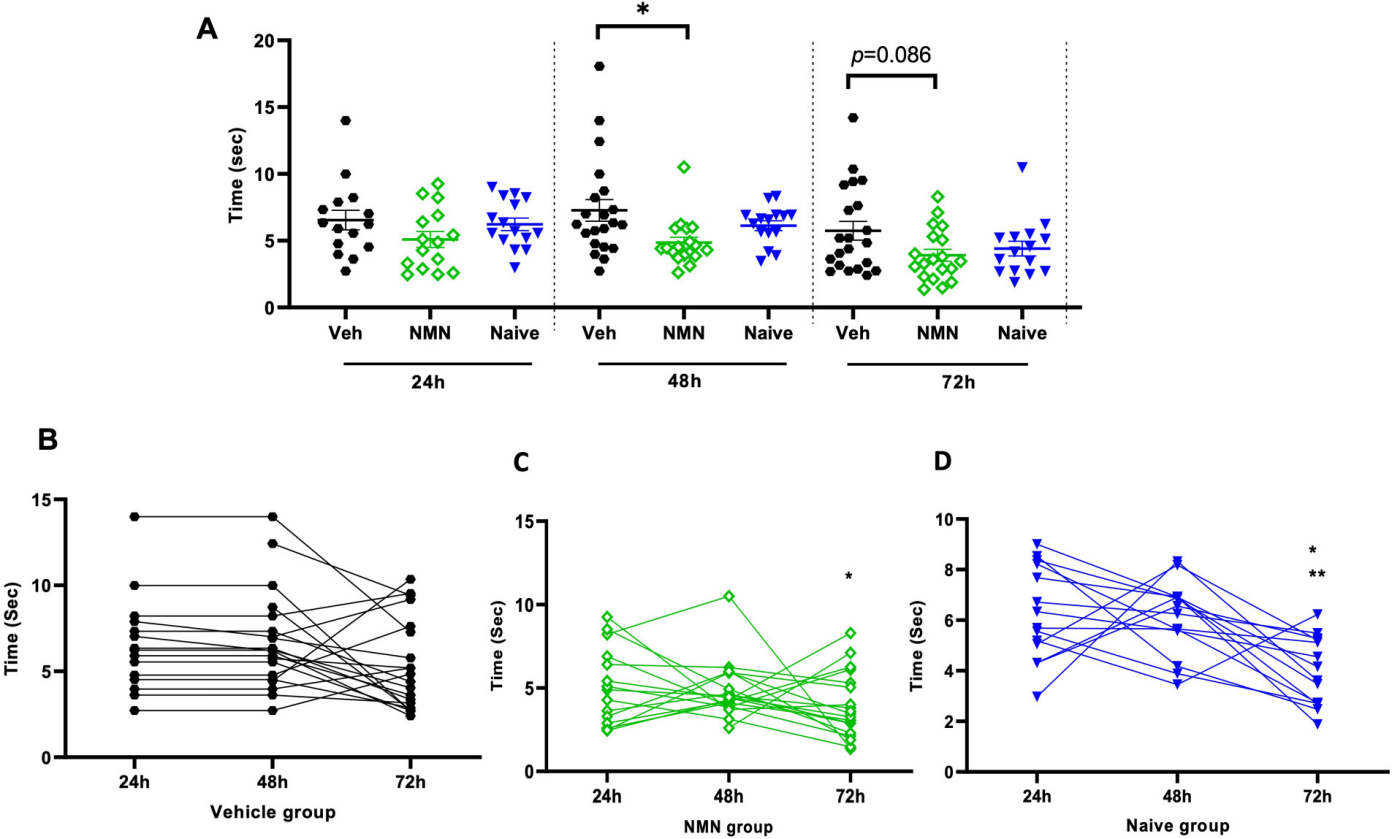
NMN effects on negative geotaxis after neonatal HI. Postnatal day 9 pups were exposed to 50 min hypoxia–ischemia (HI) with NMN treatment or without (Veh). Pups were tested for negative geotaxis at 24 h, 48 h, and 72 h after HI/NMN or HI/Veh (n = 21–18/grp/time point) and in naïve P9-P11 animals (n = 15/time point) (A). Within-group pairwise analysis of negative geotaxis performance over time in Veh-treated (B) and NMN-treated (C) HI and in naïve (D) animals. Data presented as mean ± SD. *p < .05; **p < .01.

At 24 h and 48 h after HI, the righting reflex was delayed in vehicle- compared to NMN-treated HI animals (*p *= .006 and *p *= .013, respectively; Figure 3A). In the negative geotaxis test at 48 h following HI, vehicle-treated animals demonstrated longer time to turn around on a tilted board compared to NMN-treated animals (*p *= .032; Figure 4A). Neither the righting reflex nor the negative geotaxis test was different between treatment groups at 72 h after HI (Figures 3A and 4A). Pairwise comparison data for righting reflex within groups indicated a shorter time of righting at 72 h after HI compared with 24 and 48 h in all groups (*p *< .05; Figure 3B to D). For the geotaxis test, in the vehicle group, we found no significant difference in time to turn at 72 h compared with 24 and 48 h after HI (Figure 4B), while in the NMN group, the time to turn was significantly shorter at 72 h compared with 48 h (*p *= .019; Figure 4C), and in the naïve group, the time to turn was significantly shorter at 72 h compared with both 48 and 24 h (*p *< .05; Figure 4D).

**Figure 5.**
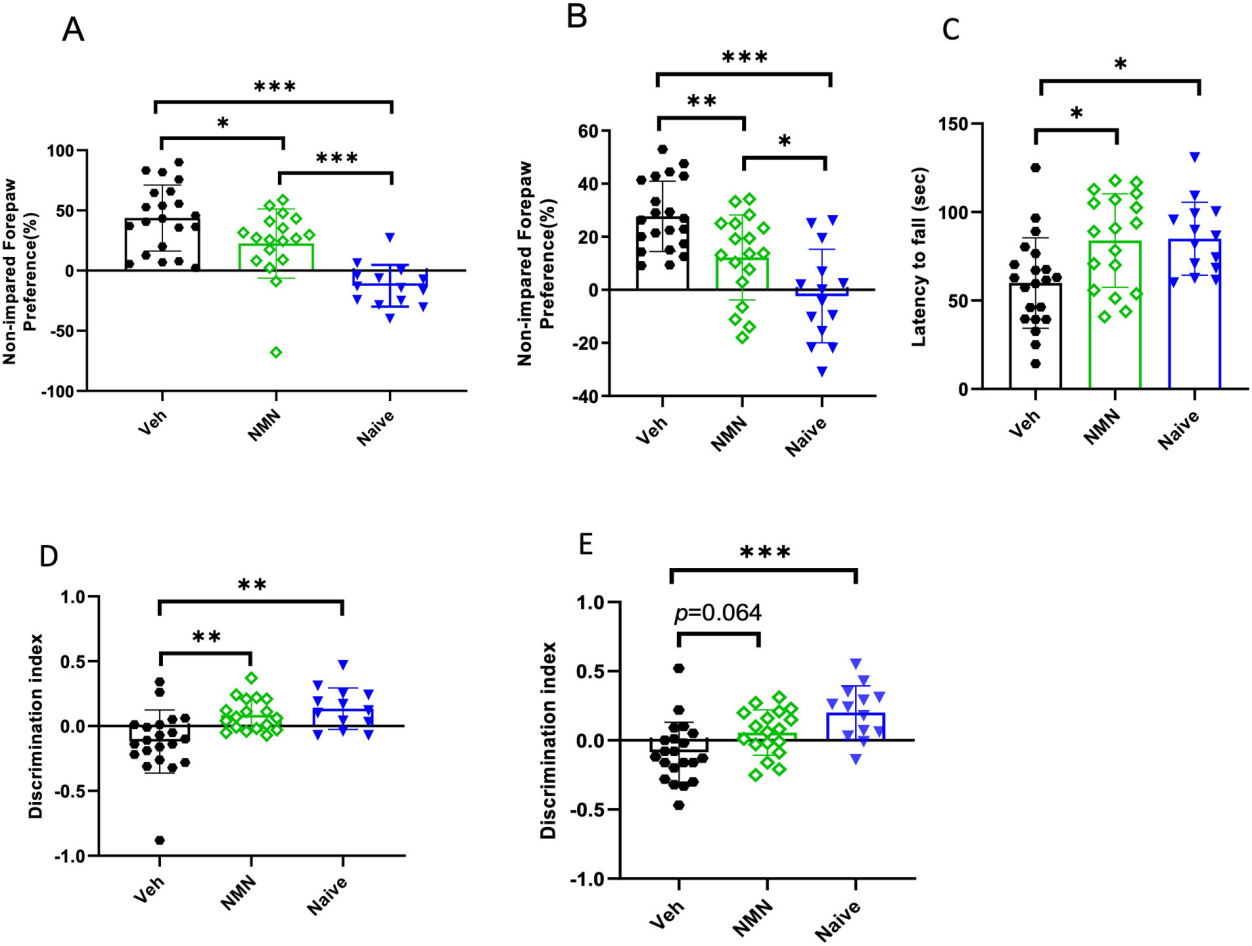
NMN effects on motor and memory function after neonatal HI. Postnatal day 9 pups were exposed to 50 min hypoxia–ischemia (HI) with NMN treatment (50 mg/kg) or without (Veh) (*n* = 18–21/treatment) and naïve animals (*n* = 14). Animals were tested in the cylinder test at P20 (A) and P45 (B) and for latency to fall in the rotarod test at P45 (C). Short-term (10 min) (D) and long-term (3 h) (E) memory was evaluated using the novel recognition test at P45 ± 5 days of age. Data presented as mean ± SD. *p < .05; **p < .01; ***p < .001.

To assess motor asymmetry after HI, left–right paw preference was evaluated using the CRT at P20 and P45 ± 5. Naïve animals showed no preference between the right and left paws in the CRT (Figure 5A and B). However, vehicle-treated mice preferred to use their left paw after HI at both P20 and P45 ± 5. NMN treatment reduced the preference for using the left paw compared to the vehicle group following HI at both P20 and P45 ± 5 (*p *= .035 and *p *= .008, respectively; Figure 5A and B).

To evaluate motor coordination, mice at the age of P45 ± 5 were assessed in the accelerated rotarod test. The latency to fall significantly decreased in vehicle-treated HI animals compared to the naïve mice (*p *= .013; Figure 5C), and NMN treatment improved motor function following HI (*p *= .011; Figure 5C). There was no significant difference between NMN-treated HI animals and the naïve group (Figure 5C).

The hippocampus is important for memory function. Therefore, we used NORT at P45 ± 5 to test NMN therapeutic effect on short-term (Figure 5D) and long-term memories (Figure 5E). Neonatal HI in vehicle-treated animals, compared to naïve animals, significantly decreased the innate preference to explore a novel object in both the short and long term (*p *= .001 and *p *= .000, respectively; Figure 5D and E). NMN treatment normalized the preference to explore a novel object both in the short and long term, and no significant difference was found between NMN-treated and naïve animals (*p *= .064; Figure 5D and E). There was no significant difference in long-term memory between NMN-treated and vehicle-treated animals (*p = *.064; Figure 5E).

### NMN Reduces Brain Injury Long Term After Neonatal HI

**Figure 6.**
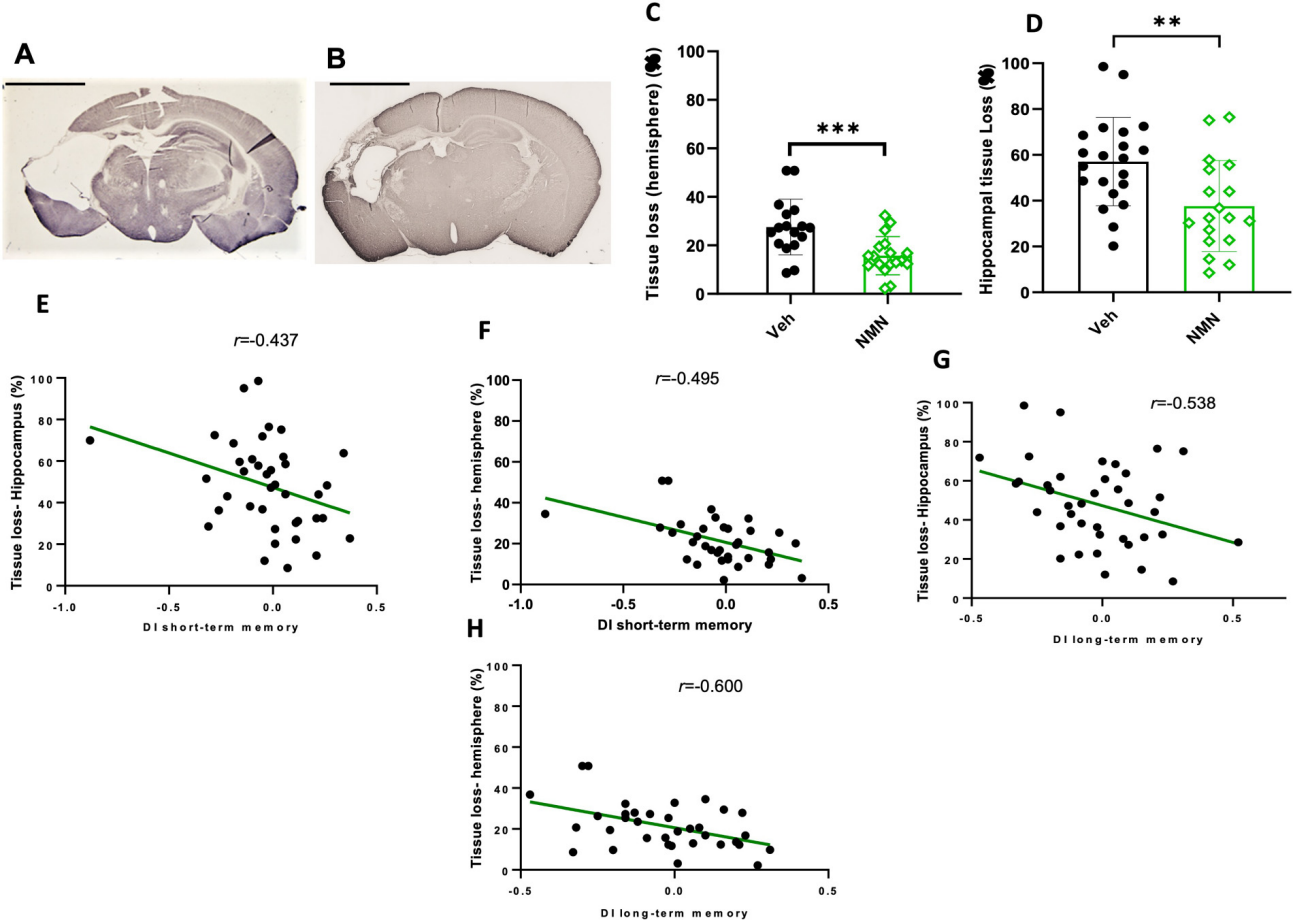
NMN effects on brain tissue loss 40 days after neonatal HI. Postnatal day 9 pups were exposed to 50 min hypoxia–ischemia (HI) with NMN (50 mg/kg) treatment or without (Veh). Examples of MAP2 staining in HI/Veh-treated (A) and HI/NMN-treated (B) animals, scale bar = 2.5 mm. The percentage of tissue loss in the ipsilateral compared to the contralateral hemisphere was calculated from MAP2-stained sections for the whole ipsilateral hemisphere (C) and for the hippocampus (D). Correlation analysis between short-term memory and tissue loss in the hippocampus (E) and tissue loss in the whole hemisphere (F). Correlation analysis between long-term memory and tissue loss in the hippocampus (G) and tissue loss in the whole hemisphere (H). Data presented as mean ± SD. **p < .01; ***p < .001.

In order to evaluate the long-term neuroprotective effects of NMN treatment, we measured tissue loss in P45 mice. Quantification of brain tissue based on MAP-2 staining showed significant reduction in tissue loss after HI in NMN-treated mice compared to the vehicle-treated animals in the whole hemisphere (*p *< .001; Figure 6A to C) and in the hippocampus (*p *= .004; Figure 6D). There were significant negative correlations between the short-term and long-term memory DI and tissue loss for both the whole hemisphere and the hippocampus, respectively (*r *= −.437, *p *= .001; *r *= −.495, *p *= .000; *r *= −.538, *p *= .000; *r *= −.600, *p *= .000; Figure 6E to H).

### NMN Reduces Microglia Activation in the Long Term After Neonatal HI

**Figure 7.**
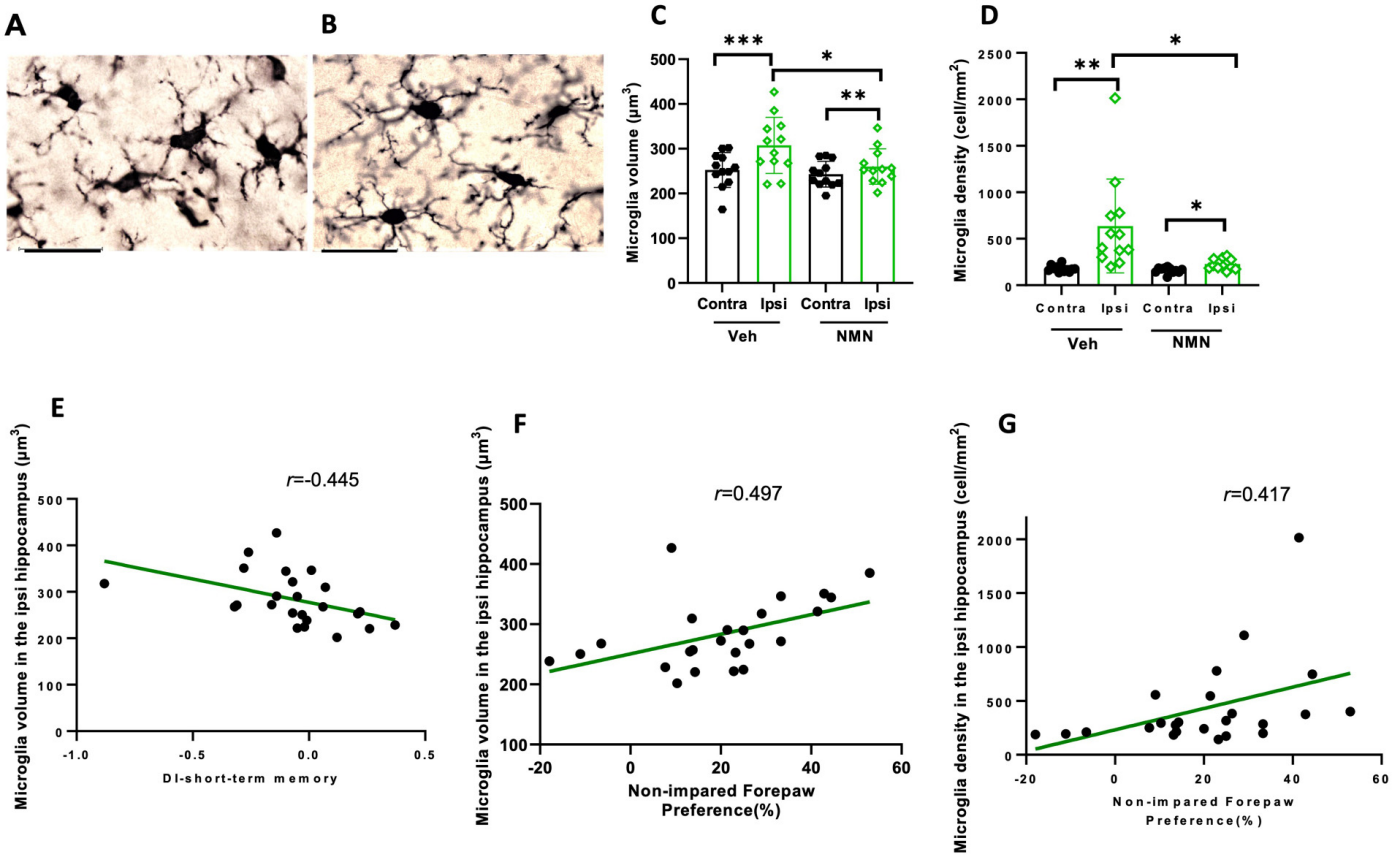
NMN effects on microglia volume and number density in ipsilateral hippocampus after neonatal HI. Postnatal day 9 pups were exposed to 50 min hypoxia–ischemia (HI) with NMN (50 mg/kg) treatment or without (Veh) and brain sections stained for Iba1 40 days after HI. Example of microglia morphology in the ipsilateral hippocampus in vehicle-treated (A) and NMN-treated (B) mice, scale bar = 30 µm. Microglia soma volume (C) and the number density of microglia (D) were determined (n = 12/grp). Correlation analysis between microglia volume in ipsilateral hippocampus and short-term memory, novel object recognition (E), and motor function, cylinder test (F). Correlation analysis between number density of microglia in ipsilateral hippocampus and motor function, cylinder test (G). Data presented as mean ± SD. *p < .05; **p < .01; ***p < .001.

In order to evaluate the long-term effects of NMN treatment on the microglia state, we analyzed microglia soma volume and number in the hippocampi of P45 mice. Volume size of microglia soma was measured as an indicator of microglia activity (Figure 7A and B). Soma size was increased in the ipsilateral side compared to the contralateral side after HI in both vehicle- and NMN-treated animals (Figure 7C), while the microglia soma was smaller in the ipsilateral hippocampus in the NMN group compared to the ipsilateral side in vehicle mice (*p *= .037; Figure 7C). To examine the effect of NMN on microgliosis, we counted the number of microglia in ipsilateral and contralateral hippocampi and found that the number density of microglia was increased in the ipsilateral compared to contralateral hippocampus in both vehicle- and NMN-treated animals, while the number of microglia was reduced in the ipsilateral hippocampus in the NMN compared to vehicle-treated animals (*p *= .017; Figure 7D). We found significant correlations between volume of microglia, short-term memory DI in novel object memory test, and nonimpaired forepaw preference (%) in cylinder test at P45 (*r *= −.445, *p *= .029; *r *= .497, *p *= .014; Figure 7E and F). There was also a significant correlation between microglia density in the ipsilateral hippocampus and nonimpaired forepaw preference (%) in the cylinder test at P45 (*r *= .417, *p *= .043; Figure 7G).

### NMN Reduces Astrogliosis Long Term After Neonatal HI

**Figure 8.**
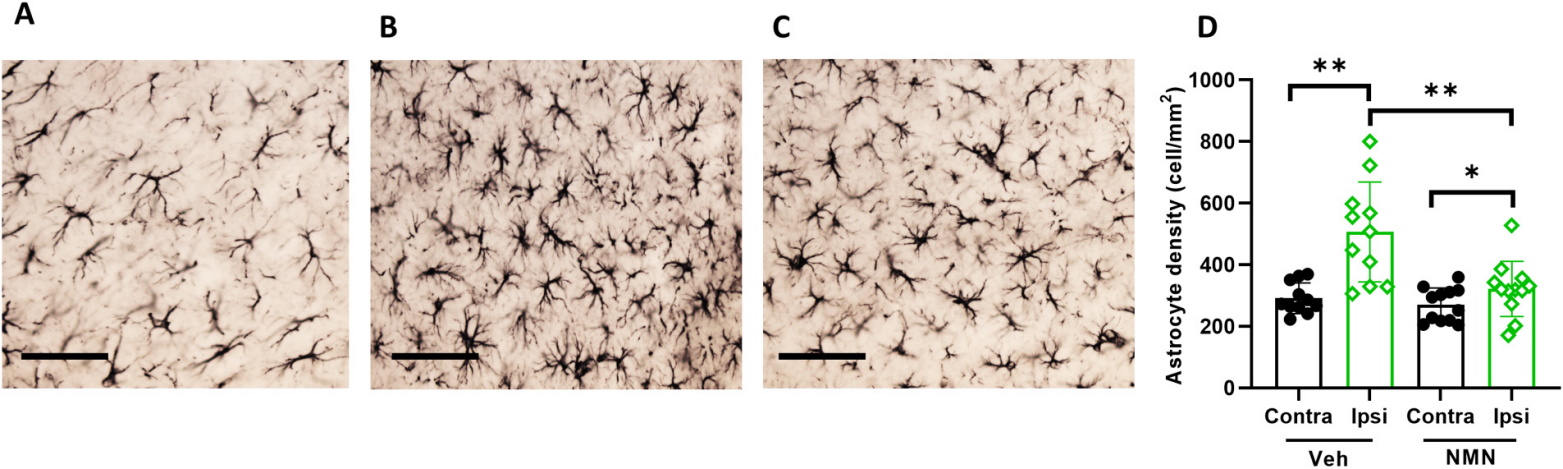
NMN effects on astrogliosis in ipsilateral hippocampus after neonatal HI. Postnatal day 9 pups were exposed to 50 min hypoxia–ischemia (HI) with NMN treatment or without (Veh) and astrocytes stained for brain sections by GFAP 40 days after HI. Examples of GFAP staining in contralateral hippocampus in Veh-treated mice (A), in ipsilateral hippocampus in Veh-treated mice (B), and in ipsilateral hippocampus in NMN-treated mice (C), scale bar = 100 µm. Astrocyte cell density was calculated (*n* = 12/grp) in GFAP-stained images. Density of astrocytes was significantly higher in ipsi hippocampus in Veh group compared to the NMN-treated mice (D). Data presented as mean ± SD. *p < .05; **p < .01.

To examine the effect of NMN on astrogliosis long term after HI, we counted the number of astrocytes in the ipsi- and contralateral hippocampi of mice at P45 (Figure 8A to C). The number density of astrocytes was significantly increased in the ipsilateral hippocampus compared to the contralateral side in both vehicle- and NMN-treated animals after HI, while the density of astrocytes was reduced in the ipsilateral hippocampus after NMN treatment compared to that in vehicle-treated animals (*p *= .004; Figure 8D).

## Discussion

In this study, we investigated the therapeutic potential of NMN on brain injury, NAD^+^ levels, SIRT1 and SIRT6 expressions, nuclear translocation of HMGB1, and astro- and microgliosis in the hippocampus in a well-established model of HI in neonatal mice. Further, NMN effects on motor and memory function were investigated following HI. We first showed that NMN at the dose of 50 mg/kg, but not 150 mg/kg or 300 mg/kg, reduced caspase-3 activity and restored hippocampal NAD^+^ concentration in the hippocampus following HI. The neuroprotective dose in our study is similar to that used to ameliorate neuronal loss in the hippocampus of adult mice following global cerebral ischemia (Park et al., 2016). In contrast, it was previously reported that a higher dose of 500 mg/kg reduced caspase-3 activity and brain weight deficit in neonatal rats (Feng et al., 2006). The reasons why a higher dose did not reduce caspase-3 activity in our study are unclear; however, it has been shown that higher levels of NMN may adversely affect injured nerves (Di Stefano et al., 2015). Further, complex dose-dependent effects of NMN on various physiological functions have been reported where a low dose of NMN (100 mg/kg/day), but not a higher dose (300 mg/kg/day), improved age-related decline (Mills et al., 2016). NMN inhibited HI-induced reduction of hippocampal SIRT6 protein expression, while hippocampal SIRT1 protein expression was not affected by HI. NMN also reduced the nuclear translocation of alarmin HMGB1 in the hippocampus. We found that NMN significantly improved early motor function from 24 h to 72 h after HI. Importantly, neonatal treatment with NMN remarkably reduced HI-induced neurological deficits in the long term (motor and memory functions), which was associated with decreased microglia activation/number and astrocyte number and attenuated tissue loss. Based on these results, we suggest that NMN, via increased NAD^+^, restores the activity of NAD^+^-dependent pathways post-HI which in turn attenuates deleterious mechanisms, which minimize neurological injury.

The level of NAD^+^ in the hippocampus of neonatal mice decreased 4 h after HI, similar to a previous study. The reason for the decrease in NAD^+^ after HI may be related to increased consumption of NAD^+^ (Ma et al., 2015). One likely pathway is via poly (ADP-ribose) polymerases (PARPs), which use NAD^+^ as a cosubstrate to PARylate target proteins. PARP activity measured by elevated PAR indicated an increase in the injured hemisphere 4 h after neonatal HI (Hagberg et al., 2004). The deacetylation activity of SIRT such as SIRT1, SIRT3, and SIRT6 consumes NAD^+^, but to our knowledge, there are no studies showing elevated SIRT activity during or after neonatal HI. The NADase or cyclic ADP-ribose synthase CD38 decreases NAD^+^ expression, and genetic deletion of CD38 shows protective effects and transient NAD^+^ decrease after ischemia in adult animals (Long et al., 2017). However, CD38 deficiency also leads to changes in the NAD^+^ metabolism pathway, which makes the direct link between CD38 reduction and protection less clear. Recent studies showed a significant effect of a single dose of NMN on the postischemic hippocampal mitochondria NAD^+^ pools (Klimova et al., 2020a, 2020b). Taken together, it is likely that elevated PARP activity contributed to the observed decrease in NAD^+^ protein expression and that changes in NAD^+^ levels may have decreased cellular damage following HI.

We found that NMN, the substrate for NAD^+^ biosynthesis, enhanced hippocampal NAD^+^ levels following HI. This result is in line with a previous study showing increased intracerebral NAD^+^ in a murine ischemic stroke model following NMN treatment (Klimova et al., 2020a, 2020b; Zhao et al., 2015). The subcellular localization of the decrease in NAD^+^ was not evaluated but is likely mostly nuclear as activation of PARP1, which causes depletion of NAD^+^, is mainly nuclear following HI in neonatal mice (Hagberg et al., 2004). However, it was recently shown that after transient ischemia in adult mice, NAD^+^ was decreased in hippocampal nonsynaptic mitochondria 4 and 24 h (but not 2 h) postischemia (Klimova et al., 2020b). These changes in NAD^+^ were reversed by a single dose of NMN immediately after ischemia. The detrimental effect of decreased NAD^+^ in mitochondria was in part mediated by a decrease in SIRT3 activity. This resulted in an increase in specific mitochondrial protein acetylation, notably of superoxide dismutase 2, which caused elevated generation of reactive oxygen species. The downstream damaging effects included mitochondrial fragmentation which was reversed by NMN treatment. More specifically, NMN reduced the fission active form of Drp1 phosphorylated at serine 616. In contrast, the levels of mitofusin (MFN) 1 and MFN2 were unchanged. It remains to be found if mitochondrial NAD^+^ is also reduced in neonatal animals after HI.

In our study, we found that the protein levels of SIRT6, but not SIRT1, were reduced in the hippocampus 12 h post-HI in neonatal mice. This finding is contrary to a previous study which showed a reduction in SIRT1 expression in the cortex and hippocampus of neonatal rats following HI (Carloni et al., 2017). A possible explanation for this might be the difference in species and severity of HI. The earlier studies used rats, and HI exposure was more prolonged (2.5 h of nitrogen–oxygen mixture, 92% and 8%, respectively), while in the current study, mice were exposed to 10% O_2_ in N_2_ for 50 min. The decrease in SIRT6 may be related to oxidative stress in the recovery phase following HI, possibly initiated by decreased mitochondrial NAD^+^ and lowered SIRT3 activity (see above). In a study using high glucose as a model for oxidative stress, downregulation of SIRT2 and SIRT6 proteins in cell lines was more severely affected than other SIRTs (including SIRT1) (Yu et al., 2016). The mechanism by which oxidative stress causes decreased levels of SIRTs, such as SIRT6, could partly involve elevated levels of MiR-34a (Baker et al., 2016). Although a change in protein level does not necessarily extrapolate to decreased enzymatic activity, reduced levels lead to lowered capacity compared to the normal situation and it has been shown that SIRT6 activity indeed is reduced by oxidative stress (Hu et al., 2015). These findings in combination with the lower availability of the cofactor NAD^+^ may contribute to a reduction in SIRT6 expression as well as reduced activity in the hippocampus after HI in neonatal mice. In line with our results, the neuronal expression of SIRT6 protein was reduced in the cortex and striatum after MCAO in adult rats (Lee et al., 2013).

Downregulation of SIRT6 has been shown to greatly enhance translocation of HMGB1 from the nucleus to the cytoplasm in stroke models in adult rats (Chen et al., 2019; Zhang et al., 2016). Neurons are the main cells which show nuclear translocation of HMGB1 following ischemic brain injury (Liu et al., 2007). Changes in HMGB1 after HI have been observed in earlier studies in neonatal mice, rats, and fetal sheep (Chen et al., 2019; Sun et al., 2019; Zhang et al., 2016), demonstrating nuclear intracellular translocation immediately after HI, while HMGB1 release to the extracellular space started several hours later. Our results corroborate these findings by showing significant reduction in cytoplasmic HMGB1 + pyramidal cells in CA1 and CA3 subregions of hippocampus in the NMN group 12 h after HI. Although extracellular levels were not determined in the current study, the decreased HMGB1 translocation by NMN suggests decreased extracellular release which may have contributed to the neuroprotection observed in our study of neonatal HI. Cellular translocation of HMGB1 has been suggested as a possible early marker of brain injury in the adult (Zhang et al., 2011), which may also be interesting to investigate in more detail in neonatal animal models.

An important finding in our study is that the increased tissue preservation following NMN treatment in mice after neonatal HI was associated with both improved early developmental behaviors and motor and memory functions at 6 weeks of age, suggesting long-lasting beneficial effects of NMN treatment. The development of the nervous system is reflected by maturation of neurological reflexes and motor coordination, and impairment of these functions following neonatal HI has been shown to correlate with brain infarct size (Ten et al., 2003). Brain injury size was also related to long-lasting learning and memory impairment following HI in neonatal rats (Ikeda et al., 2001), while other studies demonstrated a moderate relation between sensorimotor function and the severity of the brain damage 5–6 weeks after neonatal HI (Bona et al., 1997).

The protective effects of NMN are likely multifactorial including effects on SIRT (Klimova & Kristian, 2019). Based on earlier studies (Hagberg et al., 2004), we hypothesize that NAD^+^ depletion, primarily attributed to nuclear PARP1 activation following HI, results in decreased level and activity of SIRT6, which in turn lead to hyperacetylation of HMGB1 followed by its translocation to the cytoplasm and later release from the damaged cells. Although the mechanisms leading to nuclear translocation and potential release of HMGB1 in our study were not investigated in detail, the effect of restored SIRT6 activity by NMN suggests a direct link to reduced HMGB1 translocation, similar to that reported earlier in adult rats following MCAO (Lee et al., 2013). In support, the importance of HMGB1 in the development of brain injury after HI in neonatal rodents was demonstrated by the use of the HMGB1 blocker glycyrrhizin which reduced HI-induced brain damage (Le et al., 2020; Sun et al., 2019). Further, we found that the increase in neuroinflammation (astrocytes and microglia) in the hippocampus following HI at age P45 was alleviated by the administration of NMN. Future studies should clarify the underlying mechanisms that may couple NMN to decreased translocation and release of the alarmin HMGB1.

## Conclusion

This study shows that NMN improves neurological function in the long term and protects against neonatal HI-induced neuronal cell death, reduces neuroinflammation, restores the levels of NAD^+^ and SIRT6, and reduces the nuclear translocation of the alarmin HMGB1 in the hippocampus of mice. These findings have significant implications for the understanding of post-HI signaling in neonatal mice and suggest NMN as a possible novel treatment target for neonatal HI injury. However, future studies demonstrating the benefit of NMN in a HI therapeutic hypothermia model will be necessary in order to translate our current findings to practice.

## Supplemental Material

sj-docx-1-asn-10.1177_17590914231198983 - Supplemental material for Therapeutic Effect of Nicotinamide Mononucleotide for Hypoxic–Ischemic Brain Injury in Neonatal Mice Click here for additional data file. Supplemental material, sj-docx-1-asn-10.1177_17590914231198983 for Therapeutic Effect of Nicotinamide Mononucleotide for Hypoxic–Ischemic Brain Injury in Neonatal Mice by Takuya Kawamura, Gagandeep Singh Mallah, Maryam Ardalan, Tetyana Chumak, Pernilla Svedin, Lina Jonsson, Seyedeh Marziyeh Jabbari Shiadeh, Fanny Goretta, Tomoaki Ikeda, Henrik Hagberg, Mats Sandberg and Carina Mallard in ASN Neuro

## Abbreviations

ASF: = area sampling fraction
AMC: = 7-amino-4-methyl-coumarin
BCA: = bicinchoninic acid protein assay
CA1.P: = CA1 pyramidal cell layer
CA3.P: = CA3 pyramidal cell layer
CRT: = cylinder rearing test
DAB: = 3,3-diaminobenzidine
DI: = discrimination index
DoF: = degrees of freedom
GFAP: = glial fibrillary acidic protein
HI: = hypoxic-ischemic
HIE: = hypoxic-ischemic encephalopathy
HMGB1: = high mobility group box-1
HPLC: = high-performance liquid chromatography
IBA1: = ionized calcium binding adaptor molecule 1
MAP-2: = microtubule associated protein 2
MCAO: = middle cerebral artery occlusion
MFN: = mitofusin
NAD^+^: = nicotinamide adenine dinucleotide
NAMPT: = nicotinamide phosphoribosyltransferase
NMN: = nicotinamide mononucleotide
NORT: = novel object recognition test
P: = postnatal day
PARP: = poly (ADP-ribose) polymerase
RT: = room temperature
SIRT: = sirtuin.
