# Supplementary figures and images for: Therapeutic Effect of Nicotinamide Mononucleotide for Hypoxic–Ischemic Brain Injury in Neonatal Mice
Source: ASN Neuro. 2023 Oct 3;15:17590914231198983. doi: 10.1177/17590914231198983 (PMC10548811; doi:10.1177/17590914231198983)

**Supplementary Figure 1. Schematic illustration of study design**


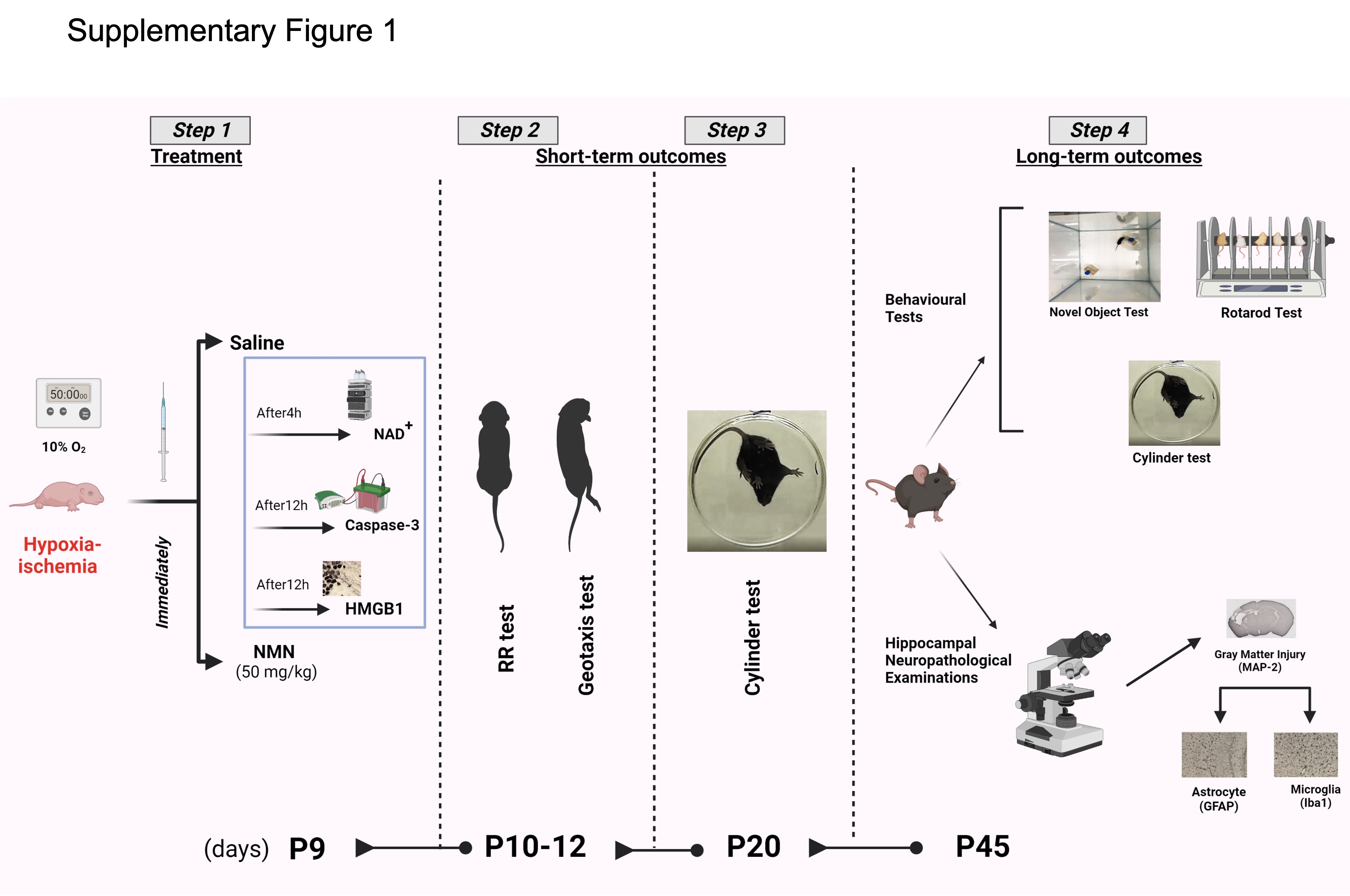

Supplement: sj-docx-1-asn-10.1177_17590914231198983 - Supplemental material for Therapeutic Effect of Nicotinamide Mononucleotide for Hypoxic–Ischemic Brain Injury in Neonatal Mice [file sj-docx-1-asn-10.1177_17590914231198983.docx]
